# Case of acute pneumoconiosis *vs.* accelerated by silicates

**DOI:** 10.47626/1679-4435-2022-1047

**Published:** 2024-09-24

**Authors:** Paola Marin Carrasco, Hector Collantes Lazo, Jonh Maximiliano Astete-Cornejo

**Affiliations:** 1 Instituto Nacional de Salud Ocupacional, La Paz, Bolivia; 2 Unidad de Medicina Ocupacional y Medio Ambiente, Universidad Peruana Cayetano Heredia, Lima, Peru

**Keywords:** pneumoconiosis, occupational disease, silicosis, neumoconiosis, enfermedad ocupacional, silicosis

## Abstract

Silicosis is an occupational lung disease caused by the inhalation of silica dust. The clinical presentation can be acute, with up to 5 years of exposure; accelerated, with less than 10 years; and chronic, with more than 10 years of exposure. The World Health Organization and the International Labor Organization include among their actions the identification of workers at risk. This study presents a case of pneumoconiosis in a worker in charge of the production of sand and clay as raw materials for other industries and who was exposed to the silicate, which has a lower concentration of silica in its composition, with an exposure time of 5 years and 11 months. The interest of this study lies in the identification of new sources of risk, since this sector very rarely reported at the national level and the need for adequate preventive measures, such as monitoring of particulate matter, engineering controls, administrative controls, and use of respiratory protection. These aspects, together with workers’ education and information, are main components in the prevention of this disease.

## Abstract (es)

La silicosis es una enfermedad pulmonar profesional ocasionada por la inhalación de polvo de sílice. La presentación clínica puede ser aguda, con 5 años de exposición; acelerada, con menos de 10 años; y crónica, con más de 10 años de exposición. La Organización Mundial de la Salud y la Organización Internacional del Trabajo incluyen entre sus acciones la identificación de trabajadores en riesgo. Se presenta el caso de una neumoconiosis de un trabajador encargado de la producción de arena y arcilla como materias prima para otras industrias que se encontraba expuesto al silicato, que tiene menor concentración de sílice en su composición, y con tiempo de exposición de 5 años y 11 meses. El interés de este estudio radica en la identificación de nuevas fuentes de riesgo, como este sector muy raramente reportado a nivel nacional y la necesidad del uso de medidas preventivas adecuadas, como el monitoreo de material particulado, controles de ingeniería, controles administrativos y uso de protección respiratoria. Estos aspectos, a los que se suman la educación e información al trabajador, son componentes principales en la prevención de esta enfermedad.

## INTRODUCTION

Silicosis is a lung disease caused by inhalation of crystalline silica particles and included in the group of pneumoconiosis. The risk of its development is related to the amount of silica inhaled throughout one’s working life. There are numerous sources of occupational exposure to inhaled silica, since its dust is present in a large number of industrial sectors. Many jobs require workers to grind, cut, drill, carve, or mill objects that release respirable aerosol containing silica particles to the environment.^1^

This study presents a case of silicosis in a worker exposed to dust generated in the production of clay and sand, which are used as raw material in ceramics, faucets, and glasses, materials that not been shown to be sources of risk for silicosis in the medical literature.

## CASE REPORT

This is the case of 34-year-old male patient, married, with complete high school education, originally from Lima, Peru, and currently living in the same city. He was a blue-collar worker with no history of pathological lung diseases or smoking, and family history was unremarkable.

### OCCUPATIONAL BACKGROUND

The patient started working informally at 21 years old as a production assistant in the unit of clay and stone quarry selection at a company in the region of Lima, for 2 years. He had a 12-hour workday, and his job consisted of moving soil containing silica (sand) daily to a hopper in a cart, in amounts of approximately from 2.72 to 3.63 m^3^, together with a coworker. This send was put into the mill built with pebble stones and was converted into a finer powder that went through a blower and a sorter, producing two materials, the finest of which was transferred to some sleeves, and the other was bagged by the production assistant, who also stacked it.

The patient was later hired by the same company as a production operator in the unit of clay and stone quarry selection to perform the same activities as those of an assistant, but added to putting the stone mill into operation, changing the sorter and blades of the blowers with 24 to 30 blades, shaking the sleeves, and driving the forklift, activities that the worker reported performing for 3 years and 11 months using a single personal protective equipment that was granted yearly.

He resigned from the job at 26 years old, after working at the company for a total of 5 years and 11 months.

### CURRENTESTATE

During medical consultation, the patient reported that, over the last years, he was qualified as unfit for work at the pre-employment medical evaluation due to changes in his chest radiograph, being rejected for jobs as a blue-collar worker. Occupational medical assessment revealed history of occupational exposure reported by the patient; physical examination found that the patient was lucid, had athletic body built, and had normal hydrated skin and mucosae. His anthropometric and clinical measurements were as follows: weight 67 kg, height 1.69 m, body mass index 23.46 kg/m^2^, breathing rate 18 breaths per minute, heart rate 76 beats per minute, blood pressure 137/73 mmHg. Furthermore, he had a flattened medial thorax, a thoracoabdominal pattern of breathing, and a vesicular murmur on auscultation, which was decreased at the infrascapular regions; the remaining physical examination was unremarkable.

### COMPLEMENTARY TESTS PERFORMED

#### Laboratory test

Blood cell count and urinalysis results were within normal parameters.

#### Radiograph

Posteroanterior chest radiograph showed no changes in soft tissues and bone parts, equidistant clavicles, regular dorsal spine margins, regular heart contours, normal diaphragm contours, free costophrenic angles, presence of hilar adenopathies, lung fields with an interstitial infiltrate consisting of small and medium rounded opacities with regular margins measuring from 0.6 to 1.8 mm in diameter, with bilateral profusion of superior, medial and inferior regions.

**Figure 1:**
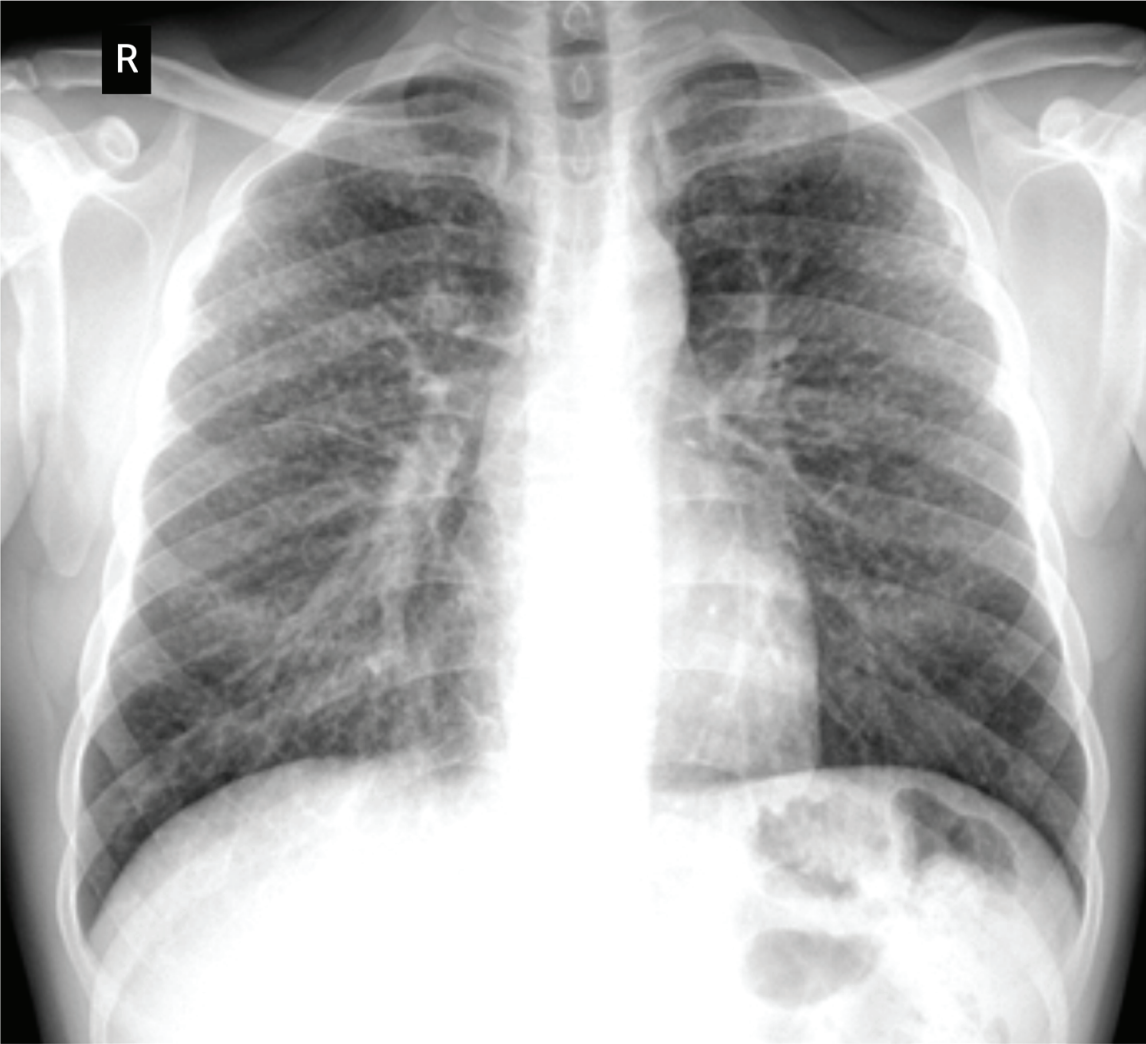
Posteroanterior chest radiograph.

Reading of the chest radiograph using the ILO International Classification of Radiographs of Pneumoconioses revised edition 2022^2^ showed a radiograph of quality grade 2 (acceptable), profusion category 3/3 q/q affecting the superior, medial and inferior regions bilaterally, with non-calcified hilar adenopathies (Figure 1).

#### Spirometry

**Figure 2:**
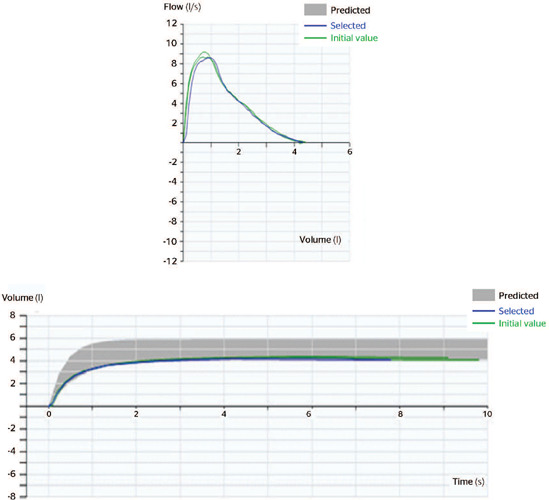
Forced spirometry: flow/volume and volume/time loops.

Patient’s spirometry met acceptability and repeatability criteria, the flow/volume loop has a sharp vertical initial section, with a triangular peak flow lasting for more than 6 seconds in the volume/time loop. Three measurements were obtained. The difference between forced vital capacity and FEV1 was lower than 150 mL; therefore, this spirometry was assigned a quality grade of A and suggested a restrictive pattern^3,4^ (Figure 2).

Due to patient’s occupational history of 5 years of exposure to silica dust and his radiographic pattern, it is concluded that this is a case of accelerated occupational pneumoconiosis resulting from silicate exposure.

## DISCUSSION

Silicon is the most frequent mineral in the Earth’s crust and is found combined with oxygen mostly, forming silicon oxides (SiO_2_, silicon dioxide) and silicates. Among the crystalline varieties of silica, the most abundant is quartz, which is present in import rocks in different proportions, with sandstone containing 100% of quartz; slate, more than 40%; and granite, 30%.^5,6^

With regard to clay, which is a raw material, it is composed mainly of silica alumina (aluminum oxide [Al_2_O_3_]), water, and variable smaller amounts of iron oxides and other alkaline materials, such as calcium oxides and magnesium oxides, all of them forming silicates.^7^

Blasting sand is the aluminum silicate product made from glazed smelting slag, which is washed, dried, and graded, produces very little dust, and is chemically neutral.^8,9^

In the present case, the apparent concentration of silica particles in an unventilated environment was able to produce a concentration of respirable pollution; consequently, the worker developed silicate pneumoconiosis. The pathogenic power of silica is related to particle size, mode and amount of inhaled silica. In the case reported here, the passage of the sand through the sorter produced respirable particles, whose inhalation was not prevented due to absence of respiratory protection equipment. In the present case, it is not possible to quantify the amount of silica dust to which the worker was exposed.^6^

Accelerated silicosis is an intermediate condition between acute and chronic forms and usually develops after 5-10 years of exposure and progresses to complicated forms of the disease more frequently and more rapidly.^10,11^

For all types of pneumoconiosis, the diagnostic process encompasses two components: documented exposure to particulate matter causing lesions and chest radiological findings.^2^ Certain diagnosis results from the simultaneous presence of occupational history of sufficient exposure to causative agents and typical radiological lesions. In the present case, occupational history revealed an exposure time of almost 6 years and occurrence of some risk factors, while chest radiograph showed images compatible with pulmonary fibrosis over the entire lung field and with profusion category III q/q, according to the ILO International Classification of Radiographs of Pneumoconioses.^2,9^

Another quantitative method to assess pulmonary involvement in pneumoconiosis is spirometry, in which lung functional capacity is affected by two mechanisms: reduced functional area for gas exchange and reduced pulmonary elasticity. Pneumoconiosis manifests basically as a restrictive disease, which presented with a mild form is in our case.^4,12,13^

Since pneumoconiosis is a preventable disease, the Occupational Safety and Health Administration (OSHA) provided a permissible exposure limit (PEL) for respirable silica of 10 mg/m^3^ divided by the percentage of SiO2 plus 2, or 250 million particles per cubic foot divided by the percentage of SiO2 plus 5, after establishing a PEL of 0.1 mg/m^3^ of respirable silica. The daily environmental exposure limit value for crystalline silica in the form of cristobalite is 0.05 mg/m^3^.^14,15^

## CONCLUSIONS

The main contribution of literature reviews on accelerated silicosis, such as that of our case, is encouraging medical evaluators to always investigate the presence of silicosis in different workplaces and sites of exposure to dust, regardless of the economic sector. In Peru, which a mining country par excellence, there is the preconception that silicosis develops only among miners; however, the present case shows that acute or accelerated cases can be found not only in mining, but also in the production of sand and clay as raw material for other industries such as construction.
